# Sialorrhea possibly associated with bupropion: a case report and review of potential mechanisms

**DOI:** 10.3389/fpsyt.2026.1979464

**Published:** 2026-10-01

**Authors:** Huijie Du, Mei Bai, Lu Yang, Shuqin Jia, Yang Yang, Wei Wang, Xiaowei Luo, Jinxuan Wei

**Affiliations:** 1 Department of Psychiatry, The First People’s Hospital of Guiyang, Guiyang, China; 2 Department of Clinical Medicine, Guizhou Medical University, Guiyang, China

**Keywords:** adverse drug reaction, bupropion, norepinephrine, quetiapine, sialorrhea

## Abstract

**Background:**

Bupropion is typically associated with xerostomia (dry mouth) rather than increased salivation. However, its prescribing information lists “increased salivation” as an infrequent adverse reaction.

**Case presentation:**

We report the case of a 59-year-old female patient with the diagnosis of comorbid anxiety and depressive disorders. The patient experienced no sialorrhea while on a combination of venlafaxine and low-dose quetiapine. However, after switching venlafaxine to bupropion, she developed significant sialorrhea. The sialorrhea was severe: she had to carry tissues and a plastic bag during the day, and at night the pillow was frequently soaked. Gradual discontinuation of quetiapine did not resolve the symptom. The sialorrhea completely remitted only after bupropion was discontinued and replaced with escitalopram. The Naranjo Adverse Drug Reaction Probability Scale score was 6, indicating a “probable” relationship.

**Conclusion:**

This case suggests that bupropion may induce sialorrhea, potentially through a synergistic mechanism involving noradrenergic pathways when combined with quetiapine. Clinicians should be aware of this rare adverse effect when prescribing bupropion, particularly in combination therapies. Therapeutic drug monitoring and clinical pharmacist collaboration may help prevent such adverse events.

## Introduction

Salivary secretion is primarily regulated by the autonomic nervous system. The parasympathetic nervous system, via acetylcholine acting on M3 muscarinic receptors, stimulates the production of large volumes of watery saliva. The sympathetic nervous system, via norepinephrine acting on β-adrenergic receptors, modulates the secretion of small amounts of protein-rich saliva. A balanced interplay between these systems maintains normal salivary flow (1, 2). Sialorrhea, defined as the excessive production of saliva or the inability to swallow saliva effectively, can lead to social embarrassment, insomnia, and risks of aspiration pneumonia. While various antipsychotics are known to cause sialorrhea, clozapine is the most notorious, with an incidence ranging from 30% to 80% (3). Other antipsychotics with similar pharmacological profiles, such as quetiapine, have also been reported to cause sialorrhea, albeit less frequently (3).

The mechanism of antipsychotic-induced sialorrhea is complex. α1-adrenergic receptor blockade has traditionally been proposed to cause oropharyngeal muscle relaxation and impaired swallowing (4). However, recent evidence points to α2-adrenergic receptor blockade as a core mechanism. Blocking α2-autoreceptors leads to a disinhibition of norepinephrine release, indirectly stimulating salivary β-adrenergic receptors and promoting the secretion of watery saliva (5). Both quetiapine and clozapine possess α2-antagonistic properties (6, 7).

Bupropion, a norepinephrine-dopamine reuptake inhibitor (NDRI), is commonly used for depression and smoking cessation (8, 9). Unlike quetiapine, bupropion is typically associated with xerostomia (10). However, bupropion SR prescribing information lists “increased salivation” as an infrequent adverse reaction, suggesting a potential direct stimulatory effect. This paradox may be explained by interindividual variability in noradrenergic tone and receptor sensitivity. Bupropion is a potent inhibitor of CYP2D6 (11), an enzyme responsible for approximately 11% of quetiapine metabolism (12, 13). Theoretically, bupropion could increase quetiapine plasma concentrations, potentially exacerbating receptor blockade effects.

Reports of sialorrhea induced by the combination of bupropion and quetiapine are scarce. This case report details a patient who developed significant sialorrhea after switching from venlafaxine to bupropion while on low-dose quetiapine. We analyze the timeline—specifically the persistence of symptoms after quetiapine withdrawal and their resolution after bupropion discontinuation—to explore the underlying mechanisms. We also discuss true secretion vs. impaired swallowing and apply the Naranjo scale.

## Case presentation

A 59-year-old female was admitted to the psychiatric department with a 9-month history of tension, excessive worry, and poor sleep.

The patient’s symptoms began 9 months prior, triggered by anxiety related to hypertension. She developed persistent worry, catastrophic thinking, anhedonia, fatigue, and somatic symptoms, including palpitations, chest tightness, and paresthesia. Previous trials of paroxetine and duloxetine had been unsuccessful. Her medical history was notable for carotid plaque, treated with atorvastatin, and hypertension, controlled with amlodipine. Importantly, there was no prior history of sialorrhea, dysphagia, or oral disease.

Physical examination revealed a blood pressure of 100/76 mmHg and a heart rate of 108 bpm. Cardiopulmonary and abdominal examinations were unremarkable. Neurological examination showed no abnormalities. Mental status examination revealed a clear consciousness and normal orientation. Speech was low in tone and rate. Mood was depressed with a sorrowful facial expression. Thought content was notable for catastrophic negative thinking, but no suicidal ideation. Cognitive functions were intact. Insight was preserved. Baseline scores: HAMA 35, HAMD 46, SCL-90 198.

The patient was diagnosed with comorbid anxiety and depressive disorders according to ICD-10 criteria. This diagnosis was supported by the severity of her symptoms: the HAMA score of 35 and HAMD score of 46 independently met the diagnostic thresholds for severe anxiety and severe depression, respectively. Organic causes of mood disturbance, such as thyroid dysfunction and structural brain lesions, were excluded by normal thyroid function tests, cranial CT, and EEG.

Given the diagnosis of comorbid anxiety and depressive disorders, the patient was initially treated with venlafaxine (an SNRI, titrated to 225 mg/day) combined with low-dose quetiapine (titrated to 100 mg/night). Lorazepam and zolpidem were used as needed. Adjunctive repetitive transcranial magnetic stimulation (rTMS) and cognitive behavioral therapy were also administered. Despite improvement in anxiety and insomnia, the patient continued to experience significant residual anhedonia and fatigue. Therefore, venlafaxine was gradually cross-tapered to bupropion sustained-release (SR, 300 mg/day) to target residual anhedonia, while quetiapine was continued. Esketamine infusions were administered three times (Aug 15, 17, 19) as an acute adjunctive treatment for severe depressive symptoms.

On August 28, 2025 (one week post-discharge), the patient developed severe sialorrhea. She had to constantly carry tissues and a plastic bag to spit out saliva, approximately 10–15 times during the day. At night, the pillow was frequently soaked, severely disrupting her sleep. The severity of the symptom was similar during the day and night. Neurological and oral examinations were normal, ruling out other organic causes. Suspecting quetiapine as the cause, it was tapered and discontinued (Aug 30 to Sep 5), and atropine sulfate 0.3 mg three times daily was added for symptomatic relief. However, the sialorrhea showed no significant change during this period, and atropine was discontinued due to lack of efficacy. Given the temporal association with bupropion initiation, bupropion was subsequently discontinued and replaced with escitalopram (20 mg/day). This intervention resulted in complete remission of the sialorrhea. (see **Figure 1** for timeline).

**Figure 1:**
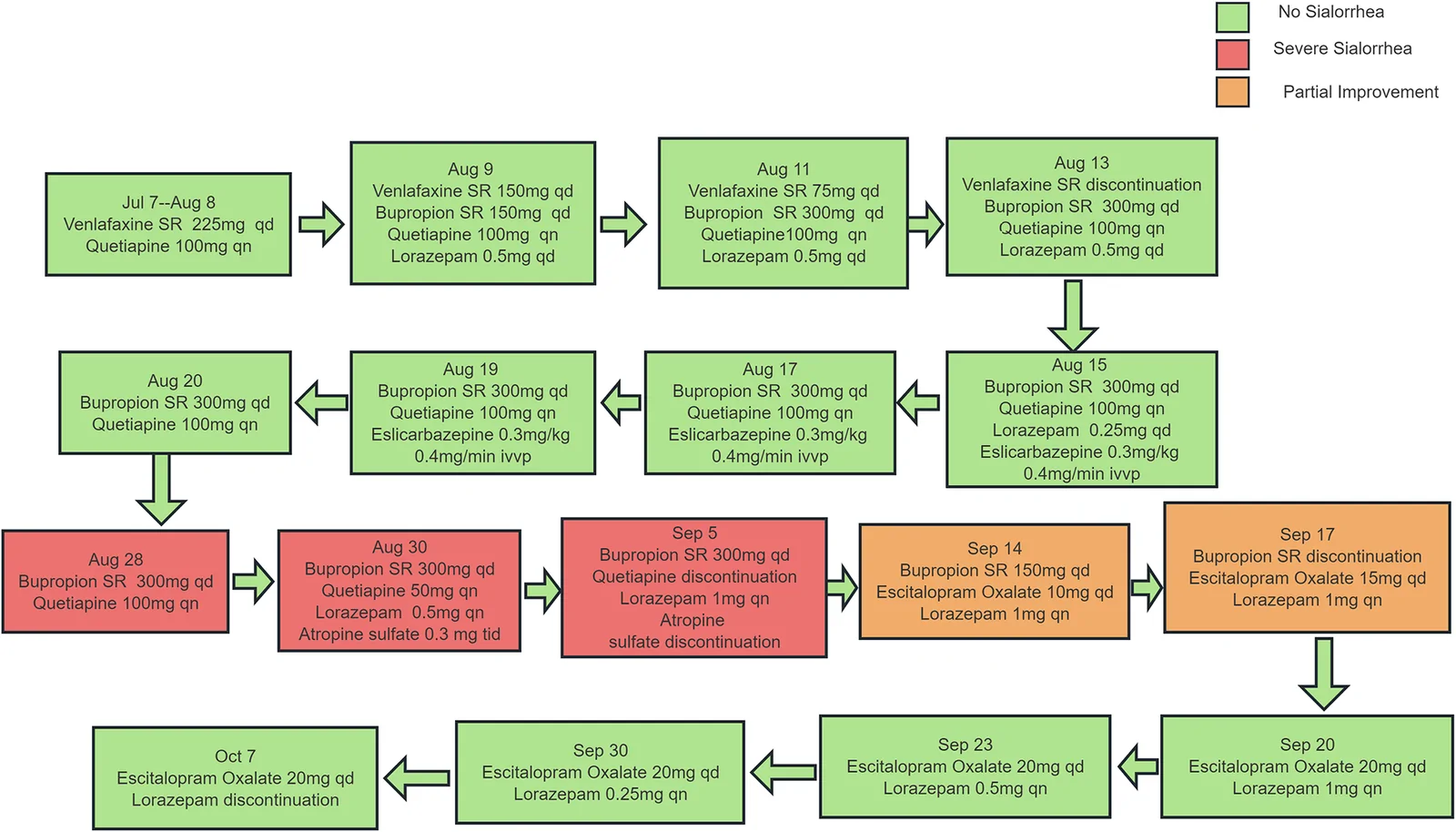
Timeline of pharmacological interventions and sialorrhea severity. Chronological timeline illustrating medication adjustments and the corresponding clinical course of sialorrhea. Color coding: Green (No sialorrhea), Red (Severe sialorrhea), Orange (Partial improvement). SR, sustained-release; qd, once daily; qn, at night; ivvp, intravenous venous pump. tid, three times daily.

The patient reported that the severe sialorrhea caused significant social embarrassment and distress. She became reluctant to go out and interact with others because she needed to spit constantly. The symptom also severely affected her sleep quality, leading to daytime fatigue and anxiety. She expressed a strong sense of helplessness when initial interventions (quetiapine discontinuation and atropine sulfate) failed to relieve the symptom. The complete resolution of sialorrhea after bupropion discontinuation brought her substantial relief and significantly improved her quality of life.

## Discussion

This case provides a unique timeline that challenges the traditional assumption that bupropion solely causes dry mouth and highlights a potential interaction with quetiapine.

### Timeline analysis: identifying the culprit

The clinical course offers decisive evidence regarding the causative agent: Phase 1 (Venlafaxine + Quetiapine): No sialorrhea. This suggests that quetiapine monotherapy (at 100 mg) or its combination with venlafaxine was insufficient to trigger the symptom. Phase 2 (Bupropion + Quetiapine): Severe sialorrhea developed. Phase 3 (Bupropion Monotherapy): After quetiapine was tapered and stopped (Sept 5), sialorrhea persisted. Given quetiapine’s short half-life (~7 hours), it would be eliminated from the system within days. The persistence of symptoms for 9 days post-discontinuation indicates quetiapine was not the sole driver. Phase 4 (Escitalopram Monotherapy): Symptoms resolved only after bupropion was stopped. This timeline suggests that while quetiapine may act as a “catalyst,” bupropion was the primary driver of the sustained sialorrhea.

### Mechanism: dual activation of noradrenergic pathways

We propose a “Dual Activation” hypothesis to explain this interaction: Quetiapine’s Role: As an α2-adrenergic antagonist, quetiapine blocks the presynaptic autoreceptors that normally inhibit norepinephrine (NE) release. This leads to an increased release of NE into the synaptic cleft. Bupropion’s Role: As an NDRI, bupropion blocks the reuptake of NE, preventing its clearance from the synapse. Synergistic Effect: The combination results in significantly elevated synaptic NE levels. Excessive NE stimulates salivary gland β-adrenergic receptors, promoting the secretion of amylase-rich, watery saliva (5).

While bupropion is a CYP2D6 inhibitor and could theoretically raise quetiapine levels, the persistence of sialorrhea after quetiapine withdrawal argues against a purely pharmacokinetic interaction. Instead, the pharmacodynamic synergy on the noradrenergic system appears to be the dominant mechanism. True Secretion vs. Impaired Swallowing: Normal neurological exam, no dysphagia, rapid resolution after bupropion discontinuation favor true increased secretion. However, impaired swallowing due to quetiapine’s α1-blockade cannot be fully excluded. Importantly, atropine sulfate produced no significant improvement, arguing against a predominantly cholinergic mechanism and supporting the noradrenergic hypothesis. Complete resolution only after bupropion discontinuation further strengthens causality.

Xerostomia Paradox: Although bupropion commonly causes xerostomia, its prescribing information lists “increased salivation” as infrequent. This may reflect interindividual variability in noradrenergic tone and receptor sensitivity, plus synergistic quetiapine effect.

### Comparison with clozapine

Interestingly, bupropion has been reported to alleviate clozapine-induced sialorrhea in some cases (14). This apparent contradiction may be due to clozapine’s unique metabolite, N-desmethylclozapine (NDMC), which acts as a partial agonist at muscarinic M1 receptors. Bupropion may inhibit the metabolism of clozapine/NDMC, altering the balance of muscarinic stimulation. Quetiapine lacks significant affinity for muscarinic receptors (15), meaning its sialorrhea mechanism is likely restricted to the adrenergic pathway described above, making it susceptible to potentiation by bupropion rather than mitigation.

### Exclusion of other factors

Esketamine: The patient received esketamine infusions, but the half-life is short (2–4 hours), and sialorrhea appeared 13 days after the last dose, making it an unlikely cause.

Benzodiazepines: Lorazepam was used but continued during the resolution phase, making it an unlikely culprit.

### Causality assessment

To systematically evaluate the likelihood of bupropion-induced sialorrhea, we applied the Naranjo Adverse Drug Reaction Probability Scale (16). The total score was 6, indicating a “probable” relationship. The scoring details are as follows: (1) previous conclusive reports of this ADR (+1); (2) the event appeared after bupropion was administered (+2); (3) the event improved when bupropion was discontinued (+1); (4) alternative causes were unlikely because quetiapine discontinuation did not resolve the symptom (+2); (5) the remaining items were scored 0 or unknown because no rechallenge, therapeutic drug monitoring (TDM), or objective laboratory evidence was available.

### Clinical implications

Although no clinical pharmacist was involved in this case, early involvement of clinical pharmacists in psychiatric care has been shown to reduce drug-related problems (17, 18). This underscores the value of integrating pharmacist expertise to prevent similar adverse events. Furthermore, future studies incorporating therapeutic drug monitoring (TDM) are warranted to verify the pharmacokinetic interaction between bupropion and quetiapine.

### Limitations

This single case report cannot establish definitive causality. The absence of TDM and CYP2D6 genotyping limits confirmation of the proposed pharmacokinetic mechanism. Although the Naranjo scale indicated a “probable” association, the proposed “dual activation” hypothesis requires further validation.

## Conclusion

This case report highlights that bupropion can induce sialorrhea, particularly in the context of noradrenergic modulation by other agents like quetiapine. Clinicians should be aware that switching from an SSRI/SNRI to bupropion may unmask or induce sialorrhea in patients taking low-dose antipsychotics. Detailed analysis of medication timelines is crucial for identifying the true causative agent in complex polypharmacy scenarios. Clinical pharmacist collaboration and TDM may help prevent such adverse events.

## Data Availability

The original contributions presented in the study are included in the article/supplementary material, further inquiries can be directed to the corresponding author/s.
